# A cross-talk between blood-cell neuroplasticity-related genes and environmental enrichment in working dogs

**DOI:** 10.1038/s41598-019-43402-4

**Published:** 2019-05-06

**Authors:** G. Guelfi, A. B. Casano, L. Menchetti, M. Bellicci, C. Suvieri, L. Moscati, P. Carotenuto, M. M. Santoro, S. Diverio

**Affiliations:** 1 Laboratory of Ethology and Animal Welfare (LEBA), Department of Veterinary Medicine, Università degli Studi di Perugia, via San Costanzo 4, 0126 Perugia, Italy; 2 Department of Surgical and Biomedical Sciences, Institution of Urological, Andrological Surgery and Minimally Invasive Techniques, Università degli Studi di Perugia, Loc. S. Andrea delle Fratte, 06156 Perugia, Italy; 3 Istituto Zooprofilattico Sperimentale dell’Umbria e delle Marche, Via G. Salvemini 1, 06126 Perugia, Italy; 4 Guardia di Finanza, Centro Addestramento e Allevamento Cani, Via Lungolago 46, 06061 Castiglione Del Lago, PG Italy

**Keywords:** Molecular biology, Neuroscience

## Abstract

This study aims to identify a panel of blood-cell neuroplasticity-related genes expressed following environmental enrichment stimulation (EE). The Drug detection (DD) training course was an excellent model for the study of EE in the working dog. This research is divided into two experimental trials. In the First Trial, we identified a panel of blood-cell neuroplasticity related-genes associated with DD ability acquired during the training course. In the Second Trial, we assessed the EE additional factor complementary feeding effect on blood-cell neuroplasticity gene expressions. In the First and Second Trials, at different time points of the DD test, blood samples were collected, and NGF, BDNF, VEGFA, IGF1, EGR1, NGFR, and ICE2 blood-cell neuroplasticity related-genes were analyzed. As noted in the First Trial, the DD test in working dogs induced the transient up-regulation of VEGFA, NGF, NGFR, BDNF, and IGF, immediately after the DD test, suggesting the existence of gene regulations. On the contrary, the Second Trial, with feeding implementation, showed an absence of mRNA up-regulation after the DD test. We suppose that complementary feeding alters the systemic metabolism, which, in turn, changes neuroplasticity-related gene blood-cell mRNA. These findings suggested that, in working dogs, there is a cross-talk between blood-cell neuroplasticity-related genes and environmental enrichment. These outcomes could be used to improve future treatments in sensory implementation.

## Introduction

Recent scientific research in learning and memory neurobiology emphasizes the existence of multiple strategies that affect neurons with short- to long-lasting functional changes through genetic regulatory mechanisms responding to neuroplasticity^1^. Neuroplasticity is a dynamic process leading to functional changes in gene expression in response to changes in environmental experience^2–4^. Neuronal plasticity allows the nervous system to learn new skills, to consolidate and retrieve memories, and to reorganize neuronal networks in response to environmental stimuli^5^.

Bloomsmith *et al*.^6^ provided a useful categorization of animal environmental enrichment (EE) as a sensorial, physical, nutritional, and sociable advancement. EE gives animals a multisensorial stimulation through the molecular epigenetic mechanism bases of neuroplasticity^7^. Recent evidence suggests how epigenetic mechanisms could create a permissive state for learning to transform experiences into long-term memories by facilitating encoding in cortical sensory processes^8^. Increasing research supports the opinion that genes related to synaptic plasticity could change in expression during a period of EE^9^. One potential mechanism by which enrichment may improve learning, memory, and synaptic plasticity, is through the regulation of transcription factor activators^10^ CREB (cAMP response element binding proteins). However, epigenetic changes behind EE persist unclearly, and the impact of EE on dog’s brain plasticity and peripheral effects remains disputed.

The mainstay of our study was the Guardia di Finanza (GdF)DD training course made available to us for investigation. For the dogs the course represents a high standard model of environmental enrichment due to the vast range of stimulations and experiences that provide cognitive challenges, social opportunities and promote the acquisition of new skills. The skills honed during training, and the intensive practice, force the dogs to adapt to EE with a related-genes remodeling “the genes learn from experience”^11^. An essential characteristic that gives value to our study, among the most salient and desirable behavioral attributes of working dogs, is the trainability and higher sensitivity to odorous molecules^12^.

To date, no scientific literature concerning canine blood-cell neuroplasticity-related genes and specific brain regions is available, and ethical reasons hinder feasible canine brain access. Recent scientific literature, however, puts forward the view that peripheral blood mRNA could be used as a biological signature for brain disorder^13–17^. Sullivan *et al*. suggest that the cautious and attentive use of peripheral gene expression may be a useful surrogate for gene expression in the CNS when it has been determined that the relevant gene is expressed in both.

Our research aims to identify, using scientific literature and bioinformatic tools, a panel of neuroplasticity related-genes, and evaluate their expression levels in the blood-cell^18^. The question, therefore, arises as to whether, and to what extent, gene expression in peripheral blood samples is comparable to gene expression in the brain^19^. In this research, we divided the experimental design into two trials, and in both, the DD training course acted as the EE factor. In the Second Trial, we introduced the complementary feeding, given during the detection training course, as EE supplementation factors. In the First and Second Trial, immediately after the DD test, we assessed blood-cell neuroplasticity-associated mRNA levels and dog behavioral evaluation of drug detection performance.

This study could provide information on the possibility of using blood cell gene expression as markers of EE effects to improve future treatments in sensory implementation. In addition, the fact that EE could modify the phenotype of blood cells is interesting *per se*.

## Materials and Methods

Experimental protocols for the study were approved by the Ethical Committee of the University of Perugia protocol number: n. 2018–21. A standing agreement between the Italian Military Force of Guardia di Finanza and the Department of Veterinary Medicine of the University of Perugia allows ethical testing on GdF working dogs.

### Animal enrolment

The dogs enrolled in the study are described in detail in the Supplementary Material S1. The dogs were physically (i.e., X-ray negative for hip dysplasia and classed in good health by a veterinarian) and behaviorally tested (i.e., the absence of behavioral pathologies identified by a veterinarian behavioral consultant) to certify their suitability for work training. It is crucial to underline that the dogs enrolled in the First and Second Trial attended the DD training course during two distinct periods but acquired the same level of expertise. For this purpose, dog-handler teams participated in a six-month DD training course at the GdF dog breeding and training center (Castiglione del Lago, Perugia, Italy). The breeding male was different in the First and Second Trial. During the treatment period, the dogs were kept in single boxes and fed with a standard maintenance diet (raw protein = 26%, crude oils, and fats = 17%, crude ash = 6.5%, crude fiber = 1.2%) and water ad libitum.

### Experimental design

The research study design was divided into two experiments called First Trial and Second Trial. Both included four sampling times: T0 - baseline value at rest, 24 hours before the search test; T1 - immediately before the search test; T2 - immediately after the search test, T3 - recovery time, 30 minutes after the end of the search test, to check if the dog had recovered baseline values. In the First and Second Trials, we assessed blood biochemical parameters (T0), heart rate (T0, T1, T2, T3), rectal temperature (T0, T1, T2, T3), DD dog performance (T2) and neuroplasticity-related gene expression profiles (T0, T1, T2, T3).

The First Trial aimed to evaluate if a panel of genes, selected by the *in silico* approach (see Supplementary Material Fig. S1), are involved in the neuroplasticity pathway activated during the DD test. In this trial, the experiment was led in one group of dogs observed at the beginning, and the end of the DD training course. The Second Trial was performed one year after the First Trial with a different group of dogs. This Trial studied only the genes correlated to neuroplasticity (according to the data obtained from the First Trial). The Second Trial was designed to assess the effect of complementary feeding on neuroplasticity mediated by changes of gene expression levels during a DD test. The experimental plane of the Second Trial involved two different groups of dogs, one fed, during the DD training course, with complementary feeding, and the other, without (control).

#### The first trial

**Figure 1:**
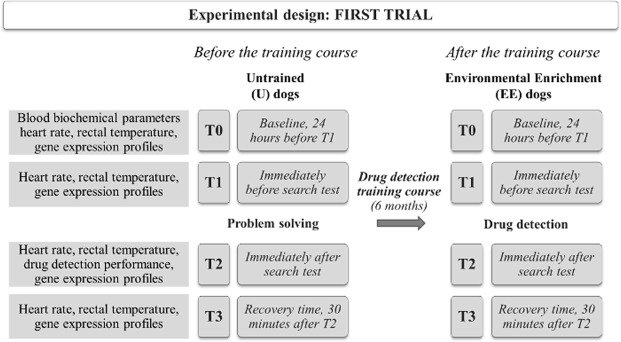
Experimental planning of the First Trial of the study. The figure schematizes the First Trial of the study that evaluates the dog gene expression levels at T1, T2, T3 in untrained dogs (U). In the First Trial, we assessed blood biochemical parameters (T0), heart rate (T0, T1, T2, T3), rectal temperature (T0, T1, T2, T3), observational examination of DD dog performance (T2) and neuroplasticity-related gene expression profiles (T0, T1, T2, T3).

In this trial, we focused on evaluating a group of dogs (n = 7), before and after the DD training course, in two different types of search tests: a problem-solving test in untrained (U) dogs, and a DD search test in trained dogs (timing and group in First Trial is illustrated schematically in Fig. 1). The DD training course included a wide range of stimulations related to different experiences, and there are well-founded reasons to view them as EE^20^, where the dogs acquired skills in DD and physical aptitudes to exercise. For this reason, we classified the dogs after the DD training course as Enriched Environmental (EE) dogs. The problem-solving test was meant to explore the innate search in skills of U dogs, while in EE dogs, it tested the skills acquired during the DD training course. U dogs carried out the problem-solving test indoors, inside the GdF warehouse. The problem-solving test consisted of finding hidden food under ten plastic cups, arranged in a horizontal line one meter from each other, within a 15-minute time-frame. At the end of the six-month DD training course, the EE dogs performed a DD search test. The search test consisted of three, 10-minute search sessions, in which the dogs had to find the drug in: (1) luggage moving on a tapis roulant simulating an airport situation; (2) different types of containers positioned on the floor where confounding substances were hidden in addition to the drug; (3) in a crowded situation, where the drug was hidden on a person (all procedures are described in detail in the Supplementary Material: Drug Detection (DD) test).

#### The second trial

**Figure 2:**
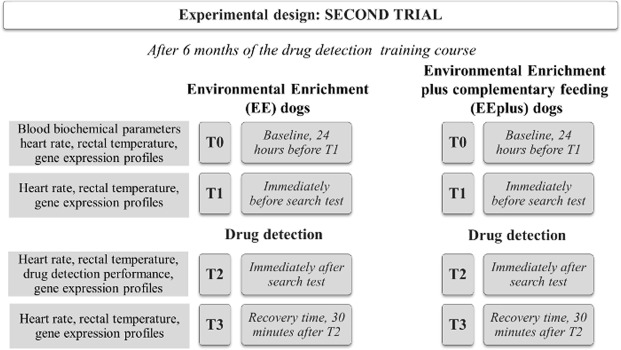
Experimental planning of the Second Trial of the study. The figure summarises two groups of dogs: one group on the left with the training course and the second group (right) with the training course plus complementary feeding. The timing and the parameters measured in the Second Trial are the same as in the First.

The dogs involved in the Second Trial attended the GdF DD training course and were trained for DD. The trained dogs, in the Second Trial, were divided into two groups: Enriched Environmental plus complementary feeding (EEplus) dogs (n = 7) that received complementary feeding during the training course and EE dogs (n = 7, as a control group) (Fig. 2). The recommended doses of Complementary feeding (IKENUP® provided by Teknofarma, Italy) were two tablets/10 kg b.w./day the first week and one tablet/10 kg b.w./day for the next 11 weeks during the DD course. One tablet of complementary feeding is composed of: L-Leucine, L-Carnitine base, L-Valine, Fructose, L-Lysine, L-Alanine, D, L-Methionine, L-Isoleucine, L-Arginine, Vitamin C, Aspartic acid, Vitamin E, Magnesium, Calcium Pantothenate, Iron, Zinc, Vitamin B_1_, Vitamin B_2_, Vitamin B_6_, Octacosanol, Vitamin B_12_ and Selenium (see Supplementary Material: Analytical content of Iken Up tablet).

### Physiological parameter and blood collection

The GdF veterinarian collected blood samples by radial venepuncture, monitored heart rate (HR) in beats per minute with a stethoscope (Classic II S.E., 3 M^™^ Littmann^®^) and measured rectal body temperature (RBT) with a digital thermometer (MB TERMO 7126500, Reckitt Benckiser SpA, Milano, Italia) at each sampling time (T0, T1, T2, T3), in each dog belonging to U, and EE (First Trial); and EE, and EEplus (Second Trial). In the First, and Second Trial, blood sampling was separated into two collection tubes: one for the analysis of biochemical parameters and one for the evaluation of gene expression profiling. Blood sampling for laboratory analysis and gene expressions are described in the Supplementary Material (Gene mRNAs and laboratory analysis blood sampling).

### Assessment of drug detection performance

Dogs and their handlers waited in an adjacent structure until called to carry out the test. The test was considered successful if/when the DD dog actively signaled the presence of the target odor by barking at his/her handler and digging in-place or on the container where the drug was hidden. The drug test was considered concluded when the handler raised his/her hand to communicate that his/her dog had signaled the presence of the target odor. The maximum time allowed to find and signal the hidden drug was 10 min when the time expired, the test was stopped and considered a miss. During the trial, an operator, situated in a nearby search area, filmed the dogs using a Digital Video Camera Recorder (Canon® MD160 and SONY® DCR-SR58, Sony®). The authors recorded the following performance data: search outcome and success/failure rate as well as detection time (in seconds) at signaling, needed for the DD dogs to find the target odor.

Next, the videos were analyzed to measure both search thoroughness and to investigate potential failure reasons in dog task performance.

### Gene expression levels of neuroplasticity-related genes: RNA isolation, reverse transcription, and qPCR amplification

The authors used Total RNA Purification kit (Norgen Biotek Corp™) to extract RNA to 100 µL of whole blood containing RNA Later, according to manufacturer instructions. RNA was quantified with the Qubit RNA assay (Life Technologies, Carlsbad, CA, USA) and stored at −20 °C until use. To ensure the purity of RNA from possible DNA contamination, we performed an additional step with DNase (RNase-Free DNase I Kit, Norgen Biotek Corp™). Total RNA (20 ng) was reverse transcribed in 20 μL iSCRIPT cDNA (Bio-Rad, Hercules, CA, USA), according to manufacturer suggestions. The authors included controls without reverse transcriptase to check for genomic DNA contamination. We carried out the qPCR amplification by using 4 μL of cDNA (diluted 1:20), 10 μL TaqMan Gene Expression Master Mix (Applied Biosystems, Foster City, CA, USA), 1 μL TaqMan Gene Expression Assays (Table 1) and water in a final volume of 20 μL. Agarose gel electrophoresis verified sample amplification fidelity. Thermocycling conditions on an iCycler iQ qPCR (Bio-Rad, Hercules, CA) consisted of an initial denaturation of 10 min at 95 °C, followed by 45 cycles of 15 seconds at 95 °C for denaturing double-stranded DNA and 1 min at 60 °C for annealing/extension steps. Bio-Rad software plotted the fluorescence intensity against the number of cycles and provided the cycle threshold (Ct) value using the automatic method. Each sample was run in triplicate and results were averaged. In parallel, to assess a genomic DNA contamination, no RT control for every RNA sample was evaluated. QPCR amplification efficiency and qPCR conditions were determined as described by Diverio *et al*.^21^. The 2^−ΔΔCt^ method was used to calculate the relative expression of the target genes^22^.

**Table 1:** TaqMan Gene Expression probe used in the studies.

Gene Symbol	TaqMan ID
EGR1	Cf02741635_m1
NGFR	Cf02697141_u1
VEGFA	Cf02674018_m1
BDNF	Cf02718934_g1
ICE2	Cf02632187_m1
NGF	Cf02697134_s1
IGF1	Cf02627846_m1
ACTB	Hs03023880_g1

### Statistical analysis

The authors chose to apply nonparametric tests because it is most appropriate with small sample sizes, and when the data does not show normal distribution. The qPCR data (2^−ΔΔCt^) were analyzed with the Kruskal-Wallis test, and the Mann-Whitney test was used to compare drug detection performance. Range, medians (Mdn) and interquartile ranges (IQR) of DD times were reported. The post hoc analyses were performed using Dunn’s post hoc test of multiple comparisons following a significant Kruskal-Wallis test. We performed both analyses with GraphPad Prism Software (GraphPad, San Diego, CA, USA) and used P < 0.05 as the cut-off for statistical significance.

## Results

### Biochemical blood parameters and physiological data

The blood biochemical parameters evaluated at T0 (24 hours before T1) First Trial (U and EE dogs) and Second Trial (EE and EEplus dogs) were comprised within the canine reference range values (Table 2). These results allow us to exclude the presence of limiting and evident dysmetabolic states that would have led to the exclusion of some subjects from the study. At T0 (First Trial and Second Trial), the mean heart rate (70–155 bpm) and rectal temperature (38.6–39.1 °C) were within the normality range, in accordance to the physiological state of the dogs (rest and work activity).

**Table 2:** Blood biochemical parameters.

Blood biochemical parameters	T0 First Trial	T0 Second Trial	Normal values
Chlorine (Cl) mmol/l	101.2 ± 3.9	109.5 ± 8.8	105–115
Ferro (Fe) μmol/l	24.9 ± 7.6	26.7 ± 9.2	15–40
Phosphorus (P) mmol/l	1.0 ± 0.19	1.37 ± 0.16	0.84–2
Glutamic Oxaloacetic Transaminase (GOT) U/l	24.5 ± 3.5	24.0 ± 5.7	23–44
Glutamic Pyruvic Transaminase (GPT) U/l	35.1 ± 11.1	37.0 ± 13.8	10–50
Alkaline Phosphatase (ALP) U/l	45.2 ± 3.2	37.6 ± 3.2	20–120
Glucose (mmol/l)	3.9 ± 0.4	5.5 ± 0.7	3.61–6.55
Lactate dehydrogenase (LDH)	174.7 ± 55.0	63.3 ± 10.6	45–233
Non-Esterified Fatty Acids (NEFA) mmol/l	0.44 ± 0.05	0.34 ± 0.05	0.4–0.7
Creatine Kinase (CK) U/l	80.3 ± 23.0	118.2 ± 34.3	30–120
Creatinine μmol/l	116.4 ± 15.5	104.5 ± 15.6	44.2–132.6

Averages ± standard deviations of blood parameters evaluated in the dogs at T0 (24 hours before T1) First Trial (U and EE) and T0 Second Trial (EE and EEplus).

### Dog behavioral evaluation of drug detection performance

All trained dogs, both in the First (EE dogs) and Second (EE dogs and EEplus dogs) Trial, detected the presence of the drug within the maximum time allowed (10 minutes). With regards to the effect of the complementary feeding, no significant differences were found in detection time. All the dogs were able to indicate where the drug sample was hidden with a detection range of 13–80 seconds for EE in the First Trial (Mdn = 46, IQR = 14–75 seconds). In Second Trial, the detection range was 13–80 seconds for EE (Mdn = 38, IQR = 16–47 seconds) and 5–86 seconds (Mdn = 45, IQR = 11–75 seconds) for EEplus. In none of the trials did the authors observe signs of stress or anxiety in the dog behavior. During the search tests, the dogs remained calm and focused on their tasks.

### Gene expression profiles of neuroplasticity-related genes

Total RNA yield was not significantly different among samples. RNA concentrations ranged from 4 to 6 ng/µl. Minimal variations in total RNA content were corrected during reverse transcription using fixed RNA input. RNA ratio 260/280 was close to 2.0 (1.8–2.0). The qPCR relative expression value was calculated by using the formula: 2^−ΔΔCt^, where ACTB is the reference gene and T0 the control gene (ΔCt target mRNA = Ct target − Ct ACTB; ΔΔCt = ΔCt target − ΔCt T0).

#### First Trial data

**Figure 3:**
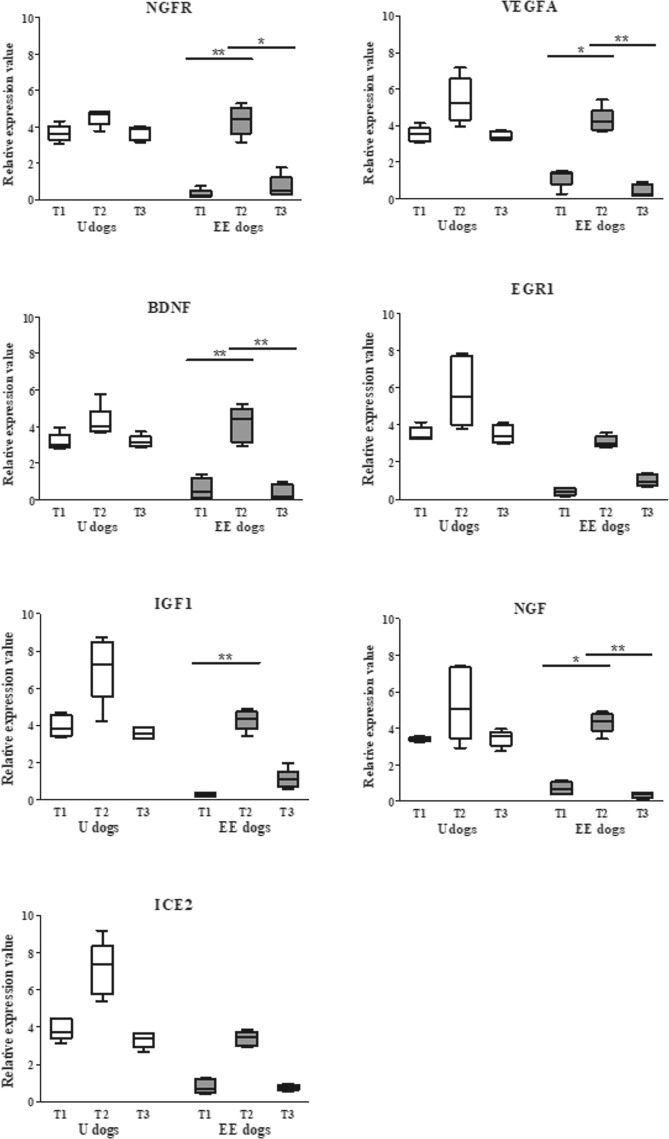
First Trial relative expression values of blood-cell neuroplasticity-related genes. The graph shows the relative expression levels at T1, T2, T3 in U dogs and EE dogs. T2 (white) represents U dogs values immediately after PS, whereas T2 (grey) indicates gene expression levels of EE dogs immediately after the DD test. The relative expression values of genes (y-axis) are presented as median (Mdn) and interquartile range (IQR) of 2^−ΔΔCt^. The EE dogs relative expression levels of genes NGFR (P ≤ 0.01), VEGF-A (P ≤ 0.05), BDNF (P ≤ 0.01), IGF1 (P ≤ 0.01) and NGF (P ≤ 0.05) showed a significant difference between T1 *versus* T2. All genes included in the study presented that the recovery time (30 minutes) was sufficient to return to T1 baseline value because there are no statistically significant differences between T1 and T3.

The qPCR data in U dogs did not show a significant difference (P > 0.05) comparing T1, T2 and T3 relative expression values of NGFR, VEGF-A, BDNF, EGR1, IGF, NGF, ICE2 genes. However, all the genes revealed an upregulation trend at T2. Conversely, five genes in EE dogs showed a significant increase in relative expression levels at T2 (immediately after the DD test) compared to T1 (P ≤ 0.05 for VEGFA, and NGF; P ≤ 0.01 for NGFR, BDNF, and IGF (Fig. 3). ICE2 and IGF1 still trended up but not significantly.

#### Second Trial data

**Figure 4:**
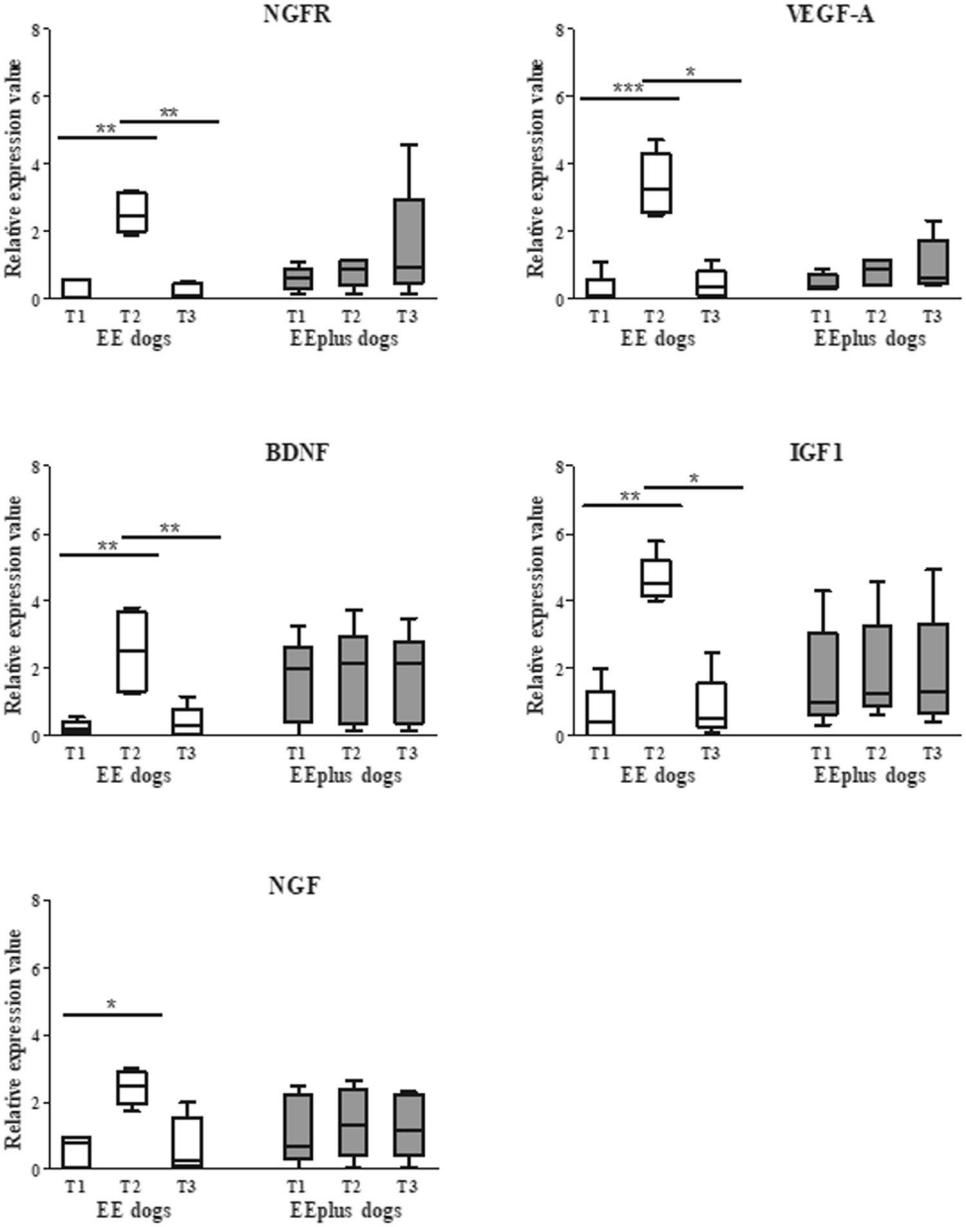
The Second Trial relative expression values of blood-cell neuroplasticity-related genes. The relative expression levels of NGFRP (P ≤ 0.01), VEGF-A (P ≤ 0.001), BDNF (P ≤ 0.01), IGF1 (P ≤ 0.01) and NGF (P ≤ 0.05) genes of EE group (white) showed a significant difference between T1 *versus* T2 (as in the First Trial). While in EEplus group (grey) relative expression values revealed no significant differences between T1, T2, and T3. All genes included in the study presented a recovery time sufficient to return to baseline.

In this trial, only the genes statistically different at T1 and T2 (First Trial) were analyzed (NGFR, VEGF-A, BDNF, IGF1, and NGF). Findings in the Second Trial highlighted that the T2 gene expression levels of EE dogs showed a statistically significant upregulation (P ≤ 0.05 for NGF; P ≤ 0.01 for NGFR, BDNF, and IGF; P ≤ 0.001 for VEGFA) as observed in the EE group of the First Trial. Conversely, in EEplus dogs, relative expression levels did not show a statistically significant difference between timepoints. Comparing T2 EE relative expression levels, and T2 EEplus, only the genes NGFR, VEGFA, and IGF1 (P < 0.01, P < 0.001, and P < 0.01 respectively) showed a statistically significant upregulation (Fig. 4).

In the First and Second Trial, when comparing T1 and T3 relative expression value, there were no statistically significant differences. The gene expression levels at T1, T2 and, T3 in EE and EEplus did not indicate significant variations.

## Discussion

This study focuses on how environmental enrichment factors directly influence the expression levels of blood-cell neuroplasticity-related genes in the working dog. The neural homeostasis is regulated by a complex neuroendocrine system involving many central and peripheral signals, but the signaling pathways regulating canine neuroplasticity are mostly unknown. Thereby, we do not assume that the mRNA found in the blood comes from the central nervous system (CNS), but that the enriched environment can influence the blood-cell^23–25^ neuroplasticity-related gene regulation as well as the CNS. Blood-cell and CNS correlation may seem more reasonable if one considers that lymphocytes often travel through many different body regions and may be exposed to the same environment as CNS tissue. This may be particularly true for the pituitary and hypothalamus (glands); having relatively direct access to the blood, they detect and respond to its changes.

Moreover, we would like to emphasize that the tissue-specific pattern of mRNA expression may indicate main clues concerning gene function. The blood transcript levels, sharing more than 80% of the transcriptome with major tissues, are exploited as EE molecular signatures in nutritional enrichment to discriminate the individual animals that benefit from nutrient supplementation^26–29^. Besides, peripheral blood mRNA expression levels are measured as a reference for monitoring an olfactory environment^30,31^. Furthermore, the use of whole blood as a surrogate for CNS expression may be permissible in the investigation of some sets of genes; it is known that on a transcriptome level, whole blood shares significant similarities in gene expression with CNS tissues^18^. The whole blood BDNF showed the same mRNA median level as CNS (excluding amygdala, hypothalamus, cerebellum peduncles^32^). No study showed a long-lasting BDNF response; therefore after EE stimulation, the basal peripheral BDNF needs to increase. In healthy humans, the brain contributes to almost 75% of circulating BDNF, suggesting that the brain is the major, but not sole, contributor to circulating BDNF while a quarter of circulating BDNF seems to stem from peripheral sources. A source of circulating BDNF mRNA/protein is the peripheral blood mononuclear cells: T and B lymphocytes^33–35^; eosinophils^36,37^, monocytes^34,35^. After the synthesis, “new BDNF” is released into the blood circulation which may, in turn, be absorbed more efficiently by central and/or peripheral tissues, where it could induce a cascade of neurotrophic and neuroprotective effects^5^. Environmental enrichment is known to exert a variety of epigenetic modifications in the brain and behavior, including neuroplasticity, improved learning, and memory. This research does not propose to evaluate which epigenetic mechanisms underlie the different regulation of gene expression, but rather the effects of environmental enrichment associated with the DD training course on circulating neuroplasticity-related genes.

The Dog Detection GdF training course ranks first in Italy and is among the best both in Europe and internationally^38^. It is based on the principle of play versus constraint in addition to favoring the animal’s instinct. The GdF drug detection training course represents an optimal environmental enrichment. Moreover, it is a learning trial that includes the acquisition of olfactory expertise and a broad range of socialization skills; this enables the dogs to move freely among crowds of people without being a risk or nuisance. An additional environmental enrichment in the GdF DD training course is the physical exercise which prepares the dogs to work with less physical effort.

In this manuscript, we examined gene levels after the drug detection training course (First Trial) and after complementary feeding given during the training course (Second Trial). We deliberately avoided a comparison between U and EE dogs because the DD training course period (6 months) is too extended to compare gene expression levels in dogs aged from 2 to 3 years old. In our opinion, during this time, a multitude of factors could induce genes to switch between activity states. In addition, we did not compare results of EE dogs-First Trial with EE dogs-Second Trial because individual differences (different parents; individual personality which could influence the way the animals perceive EE challenges) along with environmental differences (most popular drugs trafficked while the course was being held and the instructor who trained the dogs) were relevant in the analyses of a small number of dogs.

Previous studies, conducted in dogs, defined that some skills are acquired through repetitive practice to allow dogs to live in human groups^39,40^. In the DD dogs, the work skill ability has sometimes been described as a reduction in the speed of movement execution and an increase in accuracy^41^. In mice^42^ and rats, repeated exposure to an odorant could increase the olfactory receptor cells sensitivity to that odorant, due to a neuroplasticity mechanism^43^.

In the beginning of our study, and based on these theories, we hypothesized that the DD training course could teach the dogs how to search, enhancing their DD abilities through the increase of olfactory sensitivity and acquisition of skills through the mechanism of neuroplasticity. Mammalian studies have found evidence supporting a role for neurotrophic factors^44,45^ BDNF and NGF in growth and survival of 70–80% of sensory neurons^46^ during learning experiences (plasticity)^47^. The BDNF gene may be the critical protein in which transcription is enhanced during olfactory learning^48^. BDNF, NGF, NGFR and IGF genes could also be related to voluntary exercise^49,50^. Ando *et al*. (2016) studied the changes of the NGF gene in dog serum, demonstrating that epigenetic factors such as exercise, could modify NGF expression levels^51^. Regarding rodents and humans, studies have shown that exercise and intermittent fasting^52^ could enhance cognitive performance and increase serum BDNF levels^53,54^.

In the First Trial, the outcomes of our research paper showed a gene upregulation immediately after T2 only in EE dogs. This change in mRNA levels of neurotrophins NGF, BDNF, receptor NGF, growth factors VEGFA and IGF1 underlines the ability of EE dogs to activate blood-cell neuroplasticity-related genes during the DD test. Peckham *et al*.^11^ defined epigenetic dogma as the condition in which “genes learn from experience”. Conversely, gene expression in untrained dogs showed no significant difference, emphasizing that the dogs, in the absence of the DD training course, were unable to stimulate the molecular mechanism related to blood-cell neuroplasticity-related genes. To elucidate the up-regulation role of VEGF-A, BDNF, IGF, and NGF genes in EE dogs after the DD test, we need to clarify the intracellular signaling pathways that mediate gene expression. A central role in neuroplasticity has the transcription factor Cyclic AMP (cAMP)-responsive element-binding protein (CREB) because it could increase the expression of genes that modulate memory and skills^10,55,56^. CREB binding cAMP response element activated by cAMP regulates RNA polymerase activity, controls gene expression and induces transcription of growth factors (IGF-1, VEGF-A), and neurotrophins (BDNF, NGF)^10,57^. The transcriptional activity of CREB depends on its phosphorylation status and is a critical mechanism in neurogenesis regulation induced by environmental enrichment^58^. CREB could be considered as a regulator of learning, memory, and neuroplasticity^57^ in the majority of species^59–61^.

To the authors’ knowledge, there is no literature clarifying the mechanism by which BDNF, NGF, VEGFA, IGF1 and NGF receptors are not upregulated at the end of the DD test in dogs with complementary feeding in addition to environmental enrichment. Scientific evidence, in mice, shows that food intake suppresses up-regulation in genes via a BDNF-dependent mechanism^62,63^. However, it is difficult to delineate the precise effects of nutrients or bioactive food components in each epigenetic modulation because nutrients also interact with genes, other nutrients, and other lifestyle factors. Furthermore, each epigenetic phenomenon also communicates with the others, adding complexity to the system and probably, complementary feeding affected epigenetic mechanisms at multiple levels^64^.

First, nutrients act as a source of methyl groups or as co-enzymes for one-carbon metabolism that regulates methyl transfer^65^. Second, nutrients and bioactive food components could directly affect enzymes that catalyze DNA methylation and histone modifications^66^. Third, diet is the last input determining systemic metabolism which modifies cellular milieu leading to alterations in epigenetic patterns. We suppose that one of the effects of complementary feeding, used in this study, was the presence of vitamins C, E, B1, B2, B6, and B12 which reduce oxidative damage and protect reactive oxygen stress resulting in energy increase. Moreover, the reduction of oxidative stress would interact with the BDNF system leads^67^ to a loss of cell function, apoptosis, or necrosis^68^. Furthermore, soluble vitamin B has an effect on DNA methylation, carries a methyl group and delivers that methyl group for the synthesis of S-adenosyl methionine, the unique methyl donor for DNA methylation reactions^64,69,70^.

The authors hypothesize that the complementary feeding during a high metabolic consumption (detection test) in dogs, may render the BDNF up-regulation unnecessary. Energetic homeostasis and olfactory perception are linked. The feeding state modulates olfactory sensitivity in animals and humans owing to crosstalk between the feeding state and olfactory perception. The olfactory system is not merely a sensor of external chemical cues, but a parallel detector of internal chemical cues (the chemistry of metabolism)^71^.

To explain the lack of differences in the recorded DD times among the EE and EEplus dogs, we could hypothesize that the potential DD skills of the trained dogs, at the end of the training course, were expressed, in all dogs, at their maximum working capacity, therefore overriding the additional positive effect of complementary feeding. It is possible that food supplementation becomes valuable when dogs need to cope with more difficult psycho-physical conditions, such as in the case of avalanche searches and rescue situation, who often face long working periods in extreme environmental conditions. The social impact of working dogs needs a more profound understanding of all the factors that could interfere with their successful performance. This study has limitations and thus, encourages future investigations. Firstly, the size of the sample studied; although this depends on the limited number of dogs accepted in the DD training course. Besides, the First Trial used a different test for U and EE dogs; however, it was impossible to do otherwise because the dogs acquired skills only after the training course. To achieve well-defined goals, further investigation will be required to study the limiting factors concerning the performance of working dogs, including diet and health.

## Conclusion

In this study, we demonstrated that blood-cell neuroplasticity-related mRNA responds to enriched environmental signals. In the First Trial, we found a significant gene upregulation after DD. In the Second Trial, the blood-cell neuroplasticity-related genes associated with DD showed the absence of transient upregulation only in dogs with complementary feeding. Presumably, the complementary feeding allowed the dogs to carry out the test effortlessly with the same DD performance. We suppose that complementary feeding alters the systemic metabolism, which, in turn, changes BDNF and blood-cell neuroplasticity-related gene mRNA levels. The research outcomes suggest that there is a cross-talk between blood-cell neuroplasticity-related genes and environmental enrichment. The advancement of blood-cell neuroplasticity-related genes understanding should lead to improving future treatments in sensory implementation.

## Supplementary information

Supplementary Information
