# Supplementary material for: A cross-talk between blood-cell neuroplasticity-related genes and environmental enrichment in working dogs
Source: Sci Rep. 2019 May 6;9:6910. doi: 10.1038/s41598-019-43402-4 (PMC6502844; doi:10.1038/s41598-019-43402-4)
Supplement: Supplementary file 1 — Supplementary Information [file 41598_2019_43402_MOESM1_ESM.pdf]

A cross-talk between blood-cell neuroplasticity-related genes and environmental enrichment in working dogs

G. Guelfi ([1]), A.B. Casano ([1]), L. Menchetti ([1]), M. Bellicci ([1]), C. Suvieri ([2]), L. Moscati ([3]), P. Carotenuto ([4]), M.M. Santoro ([4]), and S. Diverio ([1])

Computational prediction of blood-cell neuroplasticity-related genes

The PANTHER[1–4] ontology classification system (http://pantherdb.org) was used to identify genes involved in neuroplasticity. Gene Ontology (GO) classification provides a hierarchical list of genes grouped by molecular function based on statistically significant GO terms ranked by their adjusted p-values. The seven genes selected in this study: NGF, BDNF, VEGFA, IGF1, EGR1, NGFR and ICE2 (alias NARG2 or NMDA receptor 2) presented the False Discovery Rate (FDR) <0.05. The selected genes were associated with Functional GO categories involved in neuroplasticity activity: neurotrophins family (that induce survival, development, and function of neurons[5,6]), brain-derived neurotrophic factor signaling pathway (BDNF), a regulator of the synaptic strength and plasticity[7,8], cellular response to amyloid-beta (a central component of amyloid plaques), axonogenesis, regulation and sprouting angiogenesis, negative control of cysteine-type endopeptidase activity, apoptotic process, positive regulation of cell growth, positive regulation of developmental growth, axon guidance, positive regulation of protein kinase activity.

Functional Gene Ontology categories, figure S1

Selected genes were classified into functional groups based on level 3 GO assigned as predicted for their involvement in the biological process. The percentage assigned to each GO classes are shown in Supplementary figure S1.

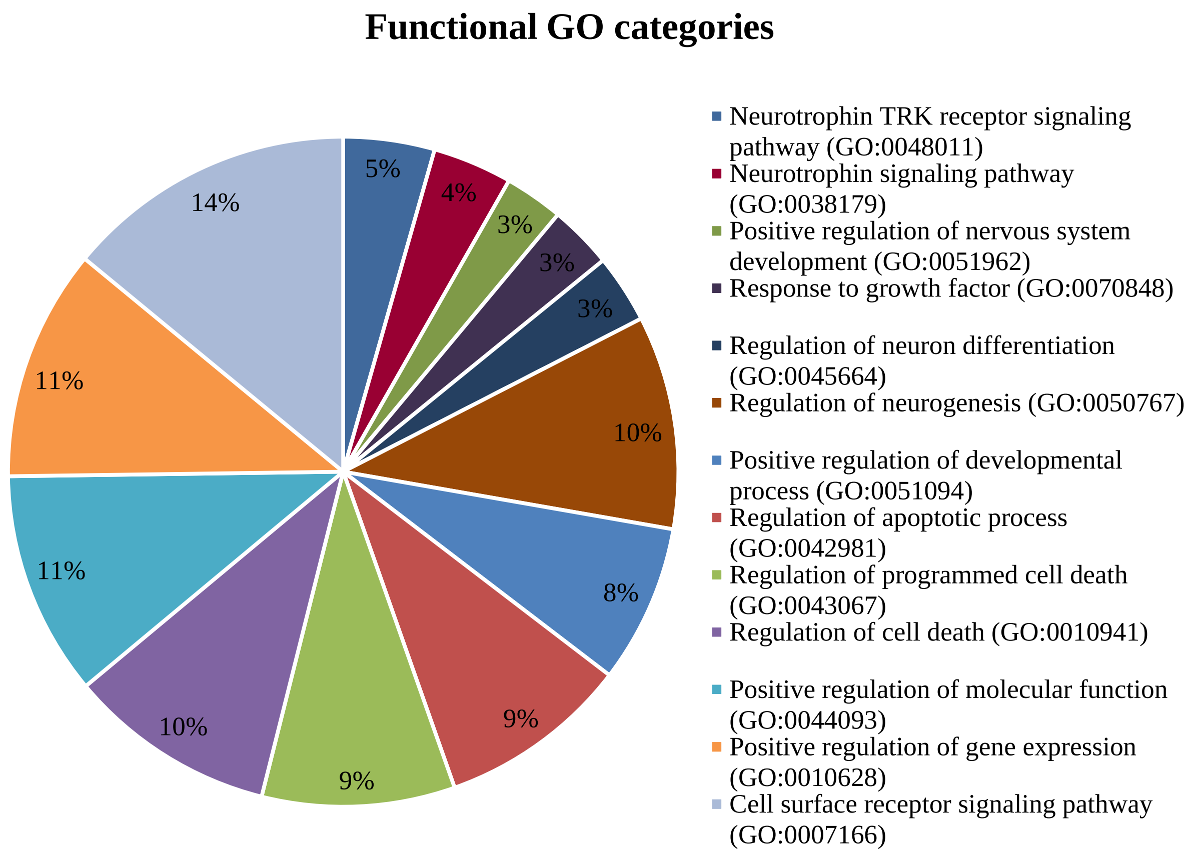

Animal enrolment statement, table S2

Identification		Number	Male	Female	Age	Weight (Kg)	Breed
**First Trial**	**U, EE**	7	3	4	2-3	23.2-30.5	German Shepherd
**Second Trial**	**EE**	7	3	4	2-3	25.0-30.0	German Shepherd*
	**EEplus**	7	3	4	2-3	25.0-30.0	German Shepherd*

* The same breeding male.

Before starting the GdF course, the dogs were selected considering the individual personality (which could influence the way which animal to perceives EE challenges) and the parents (male breeding). The First Trial used 7 “canine work units”, whereas, 14 “canine work units” were used for the Second trial. Each “canine work unit” consisted in a different team dog-handler.

All the dogs originated, were raised and trained in the same GdF dog breeding and training center (Castiglione del Lago, Perugia, Italy) where staff breeds the most suitable working dogs, guaranteeing the selection of a homogeneous bloodline.

Drug Detection test

The DD training course by the GdF dog breeding and training center (Castiglione del Lago, Perugia, Italy) ranks first in Italy and is among the best in Europe and internationally. It is based on the principle of play versus constraint (for the dog) as well as favoring the animals' natural instinct.

The drug detection test includes three 10 minutes research sessions each:

Session 1 TAPIS ROULANT,

Session 2 Wall,

Session 3 CROWD.

Session 1 was carried out in a closed environment that simulated the luggage claim area of an airport. Several pieces of luggage was positioned on the tapis roulant by an operator. The dog carried out the search operation on a leash and his handler could give him directions. During the first 5 minutes, no luggage contained the drug. After 5 minutes from the start of the test, the operator placed a suitcase containing 40 mg of cocaine (phase *TAP drug*) on the tapis roulant. The dog unit had a maximum time of 5 minutes to find the drug and complete Session 1. At the end of the session, the dog had 3 minutes of rest before the next session.

Session 2 was completed outdoors. Different types of containers (suitcases, boxes, etc.) were positioned along a wall. In some containers, confounding substances, such as food, (but no drugs) were hidden . The dog unit had 10 minutes of search time and the dog was kept on a leash. If the dog found substances different from drugs, it was considered "False signalling". The dog and his handler were given 3 minutes of recovery, after which they approached the area used for the last session.

Session 3 took place outdoors. The participants moved according to a predetermined plan which included:

- free movements with random crossing,
- disposition in line simulating lines at security checks,
- chaotic movements with random yells.

In the first step (5 minutes) no subject had drugs on him/her.

In the second step (5 minutes) a random subject had on a sample of heroine (10 g).

The dog unit had a maximum time of 5 minutes to detect the heroin. If the dog identified and reported the subject with the drug, his handler rewarded him with a 3-minute play session before starting the recovery phase.

Analytical content of Iken Up tablet

L-Leucine mg 210 - L-Carnitine base 200 mg - L-Valine 150 mg - Fructose 150 mg - L-Lysine 125 mg - L-Alanine 125 mg - D, L-Methionine 125 mg - L-Isoleucine 120 mg - L -Arginine 120 mg - Vitamin C 50 mg - Aspartic acid 50 mg - Vitamin E 50 mg - Magnesium 35 mg - Calcium pantothenate 20 mg - Iron mg 12 - Zinc 10 mg - Vitamin B1 mg 5 - Vitamin B2 mg 5 - Vitamin B6 mg 2.5 - Octacosanol µg 500 - Vitamin B12 µg 50 - Selenium µg 20.

Gene mRNAs and laboratory analysis blood sampling

Blood sampling was taken following the routine health check protocol in the DD Gdf training programme. During the GdF veterinarian procedures, the handler asked the dog to stand and stay still for 1 min, while gently manipulating and distracting it. Next, the handler asked the dog to sit, and she/he gently held it while the veterinarian collected the blood sample into vacutainer tubes (approximately 4 mL) via the radial vein.

In the First, and Second Trial, blood sampling was separated into two collection tubes: one to analyses biochemical parameters and one to evaluate gene expression profiling.

Biochemical parameters - An aliquot of T0 blood sample (3 mL) was transferred into vacuette Z Serum Sep Clot Activator (Greiner Bio-One) to evaluate the following parameters: chlorine (Cl), iron (Fe), phosphorus (P), glutamic oxaloacetic transaminase (GOT), glutamic, pyruvic transaminase (GPT), alkaline phosphatase (ALP), glucose, lactate dehydrogenase LDH, Non-Esterified Fatty Acids NEFA, creatine kinase (CK), and creatinine. The vacuette was gently inverted five times to ensure full contact with the inner procoagulant surface. The coagulation process was accomplished by maintaining the vacuette for 30 min at RT; after centrifugation (2000 ×g, 10 min), aliquots were immediately stored at −20°C until use. The concentrations of the biochemical blood parameters were measured using the automatic bio-analyser AU 400 Olympus®. The authors used the analytical methods and reagents (Beckman Coulter©) designed for the bio-AU 400 Olympus® instrument following manufacturer instructions.

Gene expression profiling - A volume (150 µL) of T0 T1, T2 and T3 blood sample was transferred into a collection tube containing 350 µL of RNA Later (Ambion, Austin, Texas, USA) and stored at

-20 °C until RNA extraction. RNA Later stabilizes and protects cellular RNA hence eliminates the need to process or freeze samples immediately.

REFERENCES

1. Mi, H., Poudel, S., Muruganujan, A., Casagrande, J. T. & Thomas, P. D. PANTHER version 10: expanded protein families and functions, and analysis tools. *Nucleic Acids Res.* **44**, D336–D342 (2015).
2. Mi, H. & Thomas, P. PANTHER pathway: an ontology-based pathway database coupled with data analysis tools. in *Protein Networks and Pathway Analysis* 123–140 (Springer, 2009).
3. Mi, H., Muruganujan, A., Casagrande, J. T. & Thomas, P. D. Large-scale gene function analysis with the PANTHER classification system. *Nat. Protoc.* **8**, 1551 (2013).
4. Mi, H. *et al.* The PANTHER database of protein families, subfamilies, functions and pathways. *Nucleic Acids Res.* **33**, D284–D288 (2005).
5. Hempstead, B. L. Dissecting the diverse actions of pro-and mature neurotrophins. *Curr. Alzheimer Res.* **3**, 19–24 (2006).
6. Reichardt, L. F. Neurotrophin-regulated signalling pathways. *Philos. Trans. R. Soc. B Biol. Sci.* **361**, 1545–1564 (2006).
7. Malenka, R. C., Nestler, E., Hyman, S., Sydor, A. & Brown, R. Molecular neuropharmacology: a foundation for clinical neuroscience. *NY McGraw-Hill Med.* (2009).
8. Huang, E. J. & Reichardt, L. F. Trk receptors: roles in neuronal signal transduction. *Annu. Rev. Biochem.* **72**, 609–642 (2003).
